# A miniaturized electronic sensor for instant monitoring of ethanol in gasohol fuel blends

**DOI:** 10.1039/c8ra02170h

**Published:** 2018-06-22

**Authors:** Muhammad Irshad, Adnan Mujahid, Adeel Afzal, Sadia Z. Bajwa, Tajamal Hussain, Waheed-uz- Zaman, Usman Latif, Muhammad Makshoof Athar

**Affiliations:** Institute of Chemistry, University of the Punjab, Quaid-e-Azam Campus 54590 Lahore Pakistan adnanmujahid.chem@pu.edu.pk +92 (0) 42 9923 0998 +92 (0) 42 9923 0463 ext. 844; Dipartimento di Chimica, Università Degli Studi di Bari “Aldo Moro” Via Orabona 4 70126 Bari Italy; Department of Chemistry, College of Science, University of Hafr Al Batin 31991 Hafr Al Batin Saudi Arabia; National Institute of Biotechnology and Genetic Engineering Jhang Road 38000 Faisalabad Pakistan; Interdisciplinary Research Centre for Biomedical Materials, COMSATS Institute of Information Technology Defense Road, Off. Raiwind Road 54000 Lahore Pakistan

## Abstract

Gasoline–ethanol (gasohol) fuel blends have gained considerable attention in the petroleum and energy sectors as relatively cheaper and greener high-octane alternative fuels with gasoline-comparable efficiency in modern transportation vehicles. However, due to different combustion rates the relative concentration of ethanol in gasohol fuel blends may vary over time. Furthermore, there is a need to monitor ethanol concentration in fuel blends for quality control applications. This article reports a miniaturized electronic sensor based on an interdigital capacitor (IDC) as the transducer and a dual-imprinted titania–polyaniline composite film as the receptor. The device has an active surface area of 0.9 cm^2^ and is easy to fabricate. The receptor material is synthesized by imprinting ethanol in both titania sol (EITS, the matrix) and polyaniline nanoparticles (EIPani, the filler), and subsequently mixing them to obtain a dual-imprinted EITS–EIPani composite. The structural and morphological characteristics of the receptor layers are determined with Fourier transform infrared (FTIR) spectroscopy and atomic force microscopy (AFM), respectively. The IDC devices are fabricated with pristine EITS and dual-imprinted EITS–EIPani composite to test their metrological sensor characteristics in standard ethanol solutions and real-time gasohol fuel blends. The instant shift in capacitance is measured upon exposure to different concentrations of ethanol. These devices show excellent sensitivity and selectivity patterns and demonstrate reliable sensor response toward ethanol in different gasohol fuel blends with 1–10 vol% ethanol. The results of this study reveal that these miniaturized ethanol sensors are potentially useful for rapid analysis of ethanol in gasohol and may be optimized for onboard fuel quality control applications.

## Abstract 2

A schematic representation of the developed setup for ethanol sensing measurements in gasohol blends.

## Graphical abstract

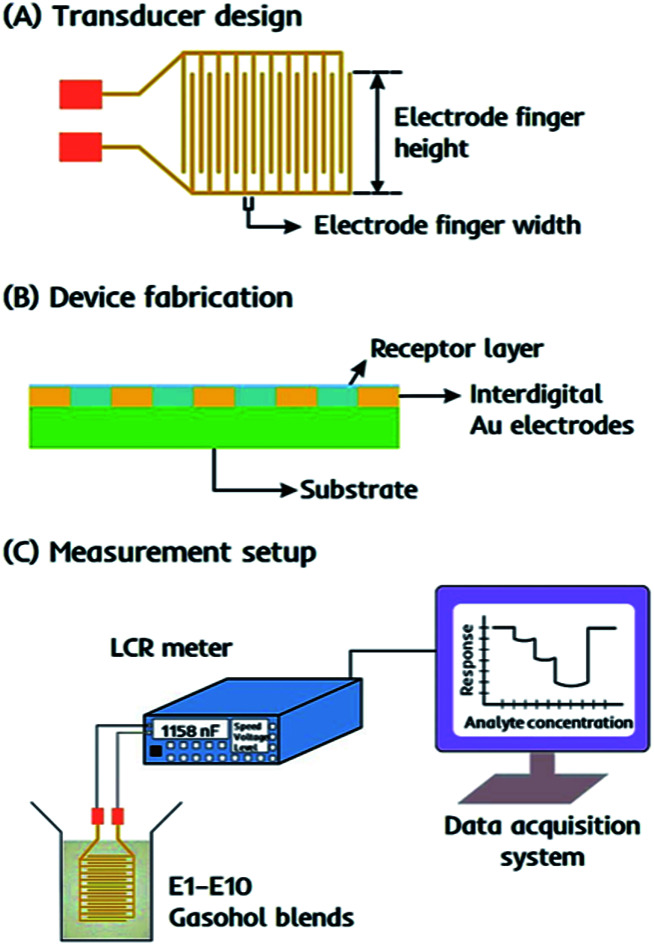

## Introduction

Petroleum and other fossil fuels are almost exclusively used worldwide for transportation and energy needs. At a time when many crude oil-importing nations are suffering economically, the major oil-producing countries are also facing a risk of rapidly depleting crude oil sources. Furthermore, the adverse environmental impact of fossil fuel consumption in the form of greenhouse gas emissions, global warming, and climate change is raising awareness and concerns internationally. This has led to a growing demand to reduce the dependency on petroleum and other fossil fuels.^1^ Therefore, major research efforts are currently centered on the discovery of renewable energy sources and alternative fuels, which are non-petroleum and are, for instance, bio-based.^2^

The alternative fuels based on biomass energy sources such as ethanol and biodiesel are extremely important and capable of gradually replacing fossil fuels.^3–6^ The physicochemical properties of ethanol and its compatibility make it suitable for use in spark ignition combustion engines. Furthermore, ethanol as fuel does not require major design alterations in modern combustion engines due to its functional similarity with gasoline (petrol). Since 1986, ethanol has been used to boost the motor octane number of gasoline.^7^ Binary gasoline–ethanol blends, often termed as gasohol, have been formed by mixing ≤10% by volume anhydrous ethanol in gasoline and have been used as transportation fuel, recognized as E10.^8–10^ It is unusual for an alternative fuel, but ethanol has been reported to potentially enhance the efficiency and performance of internal combustion engines.^11,12^

Table 1 shows the physicochemical properties^13–20^ of gasoline, ethanol, and gasohol (E10) blended fuel. The lower stoichiometric air to fuel ratio and significantly higher heat of vaporization of ethanol lead to increased adiabatic charge cooling of the stoichiometric air–E10 mixture.^19^ In addition, the emission of greenhouse gases is also influenced by ethanol. As a consequence of oxygenate (*i.e.* ethanol) blending, the formation of noxious gases such as specific CO_2_ emissions is sufficiently reduced.^21,22^ Lastly, the presence of oxygenates also reduces the particulate emissions by lowering the concentration of intermediates needed for the growth of aromatic soot particles.^23^ Thus, gasohol blends, *e.g.* E10, are advantageous in several ways as economically and environmentally better alternatives for pristine gasoline.

**Table 1:** Physicochemical properties of typical gasoline, ethanol, and gasohol (E10) fuels relative to internal combustion engines.^13–20^

Properties	Unit	Gasoline	Ethanol	E10 (Gasohol)
Source	—	Crude oil	Biomass	Gasoline–ethanol Blend
Percent by volume ethanol	%	0	100	10
Percent by mass oxygen content	%	0	34.7	3.47
Density at 15.6 °C	g cm^−3^	0.7400	0.7396	0.7890
Reid vapor pressure	psi	7–15	2.3	9.0
Stoichiometric air to fuel ratio	kg kg^−1^	14.7	9.0	14.4
Energy per unit mass of air	MJ kg^−1^	2.92	2.99	2.92
Flash point	°C	−55	12	−40
Auto-ignition temperature	°C	246	365	260
Energy density	MJ kg^−1^	42.90	26.95	41.24
Volumetric energy content	MJ L^−1^	31.70	21.30	31.25
Heat of vaporization	kJ kg^−1^	350	838	405
Research octane number (RON)	—	95.0	109.0	97.1
Motor octane number (MON)	—	85.0	89.7	85.6
Specific CO_2_ emission	kg kg^−1^	3.17	1.91	3.04

Several countries have already adapted gasohol blends as an alternative transportation fuel to reduce fossil fuel consumption, noxious emissions, and price per gallon.^6,24^ In many modern vehicles E10 is an efficient alternative for premium unleaded gasoline. However, to their disadvantage, these binary gasohol blends are consumed relatively faster due to lower energy thus resulting in 1–3% lower mileage per gallon.^25,26^ In fact, the burning velocity of ethanol is higher than gasoline, which is beneficial in terms of increasing levels of exhaust gas recirculation and decreasing throttling losses.^27^ However, this also leads to dilution and results in fluctuating ethanol concentrations. It is therefore important to monitor ethanol concentration in blended fuels. A number of analytical techniques have been used in the past to determine ethanol content in gasohol blends.^28–35^ However, these methods are inappropriate to instantly check the quality of blended fuels and monitor ethanol concentration in real-time due to sample acquisition and preparation procedures.

In this regard, the development of smart, miniaturized devices for onboard installation and instant monitoring of blended fuels in modern vehicles is a practical solution.^36^ To date, the sensors for quality control of gasohol blends are not widely developed due to complex nature of the fuel.^37–40^ Therefore, the objective of this work is to design a miniaturized electronic device for instantaneous detection and real-time monitoring of ethanol in gasohol blends. The device is composed of a miniaturized interdigital capacitor (IDC) as the transducer and coated with dual imprinted titania–polyaniline composite layer *i.e.* ethanol-imprinted titania sol (EITS) with ethanol-imprinted polyaniline (EIPani) as the receptor. The device is characterized and tested in ethanol–hexane mixtures to study its metrological characteristics. Finally, the device is exposed to different binary gasohol blends *viz.* E1–E10 containing 1–10 vol% ethanol to substantiate its potential as a reliable sensor for monitoring complex mixture. The main highlight of this study is the development of robust and inexpensive transducer *i.e.* interdigital electrodes patterned on printed circuit boards (PCBs) combined with dual imprinted composite layer as both the dispersion matrix (titania sol) and filler (polyaniline) are imprinted with ethanol. And ultimately, the designed setup could be used suitably to monitor ethanol in gasohol blends.

## Experimental section

### Materials

Aniline [C_6_H_5_NH_2_; 99.5% extra pure], *n*-hexane [C_6_H_14_; 95% anhydrous, analytical grade], and 1-propanol [C_3_H_8_O; 99%] are obtained from Riedel-de Haen. Ethanol [C_2_H_5_OH; 99.8% anhydrous] is supplied by Merck, and titanium tetrachloride [TiCl_4_; purity ≥ 98%] is purchased from Fluka. While titanium(iv) *tert*-butoxide [Ti(O-*t*-Bu)_4_; purity 97% reagent grade], and potassium dichromate [K_2_Cr_2_O_7_; 99.5%] are obtained from Sigma-Aldrich. Premium gasoline is obtained from local fuel station and is used to prepare gasohol blends with 1–10 vol% ethanol. Initially, ethanol standards are prepared in *n*-hexane by mixing 1–10 vol% of anhydrous ethanol to measure primary device characteristics. For selectivity measurements, the standard solutions of 1-propanol are also prepared in *n*-hexane. Subsequently, the gasohol blends [E1–E10] with 1–10 vol% anhydrous ethanol are prepared according to the ASTM standard D7717-11 for preparing volumetric blends of denatured fuel ethanol and gasoline blend stocks for laboratory analysis.^41^

### The receptor

**Fig. 1:**
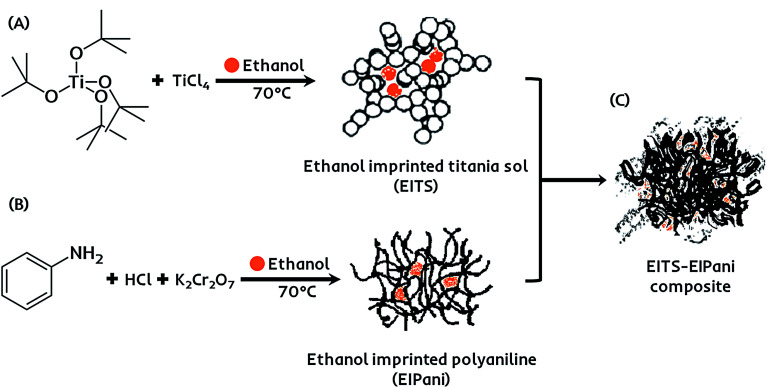
(a) Synthesis of ethanol imprinted titania sol *via* titanium(iv) chloride catalyzed hydrolysis of titanium(iv) *tert*-butoxide. (b) Synthesis of ethanol imprinted polyaniline *via* potassium dichromate initiated oxidative polymerization of aniline. (c) The formation of dual-imprinted EITS–EIPani composite *via* ultrasonic mixing of constituents.

The receptor layer for selective recognition of ethanol in gasohol blends is composed of a molecularly imprinted composite film. Imprinting of molecular targets is a straightforward and practical approach to induce sensitivity and functionality consciousness in a material of choice.^42^ Hereby, a dual-imprinting technique is applied to obtain the receptor film for the electronic sensing device. Firstly, ethanol imprinted titania sol (EITS) is prepared by mixing 670 μL of Ti(O-*t*-Bu)_4_ in 1 mL of anhydrous ethanol, which acts as the solvent as well as the template.^43^ The mixture is heated to 70 °C for 10 minutes in a water bath, and 100 μL of TiCl_4_ is added under constant magnetic stirring at 200 rpm for 1 hour. A clear and transparent sol with slightly yellowish tint is collected and stored at 5 °C. Secondly, in a separate chemical reaction vessel, a molecularly imprinted conducting polymer, *i.e.* ethanol imprinted polyaniline (EIPani), is produced by mixing 5 mL of distilled aniline in 50 mL of 2.0 M HCl under gentle stirring at 0 °C. 2 mL of ethanol is gradually added to the acidic reaction mixture. Subsequently, 10 mL of 1.0 M K_2_Cr_2_O_7_ are added drop-wise from a burette in 45 min. The mixture is kept at room temperature for 1 hour to complete the polymerization process. The resultant black color precipitates of EIPani are then vacuum filtered and are thoroughly washed with 2.0 M HCl and distilled water to remove unreacted K_2_Cr_2_O_7_ and acidic contents, respectively. Finally, 1 mg of EIPani is carefully dispersed in 100 μL of EITS using ultrasound sonication for 10 min to obtain a homogeneous dispersion. This EITS–EIPani dispersion is then used to fabricate a composite film on the device. Fig. 1 demonstrates the chemical pathway to prepare the selective receptor films composed of EITS–EIPani composite. In a similar method, non-imprinted polyaniline (NIPani) and single-imprinted EITS–NIPani composite are also prepared as reference materials to compare device performance and measure the imprinting effects.

### The transducer

**Fig. 2:**
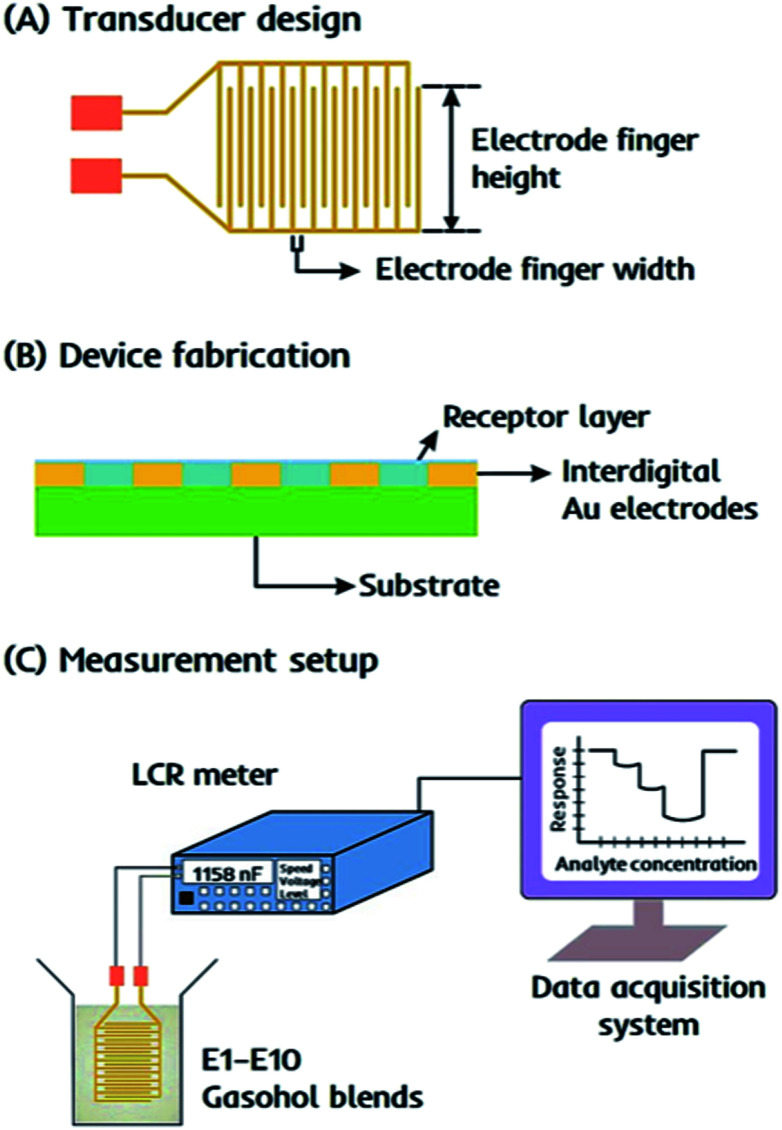
(a) An interdigital capacitor (IDC) patterned on a circuit board. The transducer has screen patterned interdigital gold electrodes with finger height: 9.0 mm, finger width: 300 μm, and spacing between two consecutive fingers: 130 μm. (b) A thin receptor layer is fabricated on the transducer surface by spin coating dual-imprinted EITS–EIPani composite. (c) A basic representation of the setup for sensing measurements in gasohol blends is shown.

The device consists of an interdigital capacitor (IDC) as the transducer that is easy to fabricate and responds instantly to the changes in electrochemical properties, *i.e.* capacitance of the receptor, when exposed to gasohol blends. An equivalent circuit for such interdigital electrodes as electrical transducer has been discussed by Reboun and Hamacek^44^ for monitoring humidity using sulfonated aluminum phthalocyanine layer. The electrical parameters measured in parallel mode *i.e.* resistance (*R*_p_) and capacitance (*C*_p_) mainly depends on the layer properties covering the electrode surface. Therefore, any change in proximity of layer material *i.e.* due to layer–analyte interactions would lead to change these parameters and thus, taken as sensor response. Molecular imprinted layers^45,46^ can be taken as efficient recognition interface for covering the electrode surface. IDCs are designed as printed circuit board pattern using screen printing method with the electrode finger width 300 μm and spacing between two consecutive figures of 130 μm. With an approximate electrode finger height of 9.0 mm, the active surface area of the device is around 0.9 cm^2^. The design details of IDC electrodes are presented in Fig. 2a.

### Characterization

The receptor materials including EITS, EIPani, and EITS–EIPani composite are characterized by Agilent Cary 630 Fourier transform infrared (FTIR) spectrophotometer to investigate the structural features, and by Shimadzu SPM-9700HT atomic force microscope (AFM) to study the microstructure and surface morphology.

### Device fabrication

The receptor layer is fabricated on the surface of an IDC transducer by spin coating dual-imprinted EITS–EIPani composite, as shown in Fig. 2b. Before coating, the transducer surface is cleaned with distilled water and acetone, respectively. The dual-imprinted EITS–EIPani composite is sonicated in an ultrasound sonication bath for 10 min to form a uniform dispersion. Subsequently, 20 μL of the respective dispersion is spin coated on IDC surface at 2000 rpm for 2 min. After coating, the receptor layer is dried under vacuum at room temperature to remove entrapped ethanol molecules. Multiple devices are also fabricated by spin coating EITS, EITS–EIPani and EITS–NIPani composite film to study relative sensor performance.

### Sensor measurements

Sensor measurements are recorded as the shift in electrochemical properties of the receptors, *i.e.* capacitance of the sensing element by Sourcetronic ST2817B LCR meter. The devices coated with different receptor layers are exposed to standard ethanol solutions, *i.e.* 1–10 vol% ethanol in *n*-hexane, stored in airtight vials, and the resultant change in capacitance is monitored in parallel mode. For all measurements, 50 Hz is selected as the optimal frequency to obtain the highest shift in the sensor signal. The basic experimental setup for sensor measurements is shown in Fig. 2c. All measurements are carried out under the following experimental conditions: voltage 0.3 V, frequency 50 Hz, speed-medium, range (auto). The sensor responses are also recorded for standard 1-propanol solutions, *i.e.* 1–10 vol% propanol in *n*-hexane to study device selectivity. Finally, the devices are exposed to real-time gasohol samples E1–E10 containing 1–10 vol% ethanol in gasoline blend stocks under similar experimental conditions. All measurements are recorded at 25 °C.

## Results and discussion

### Structural characterization

**Fig. 3:**
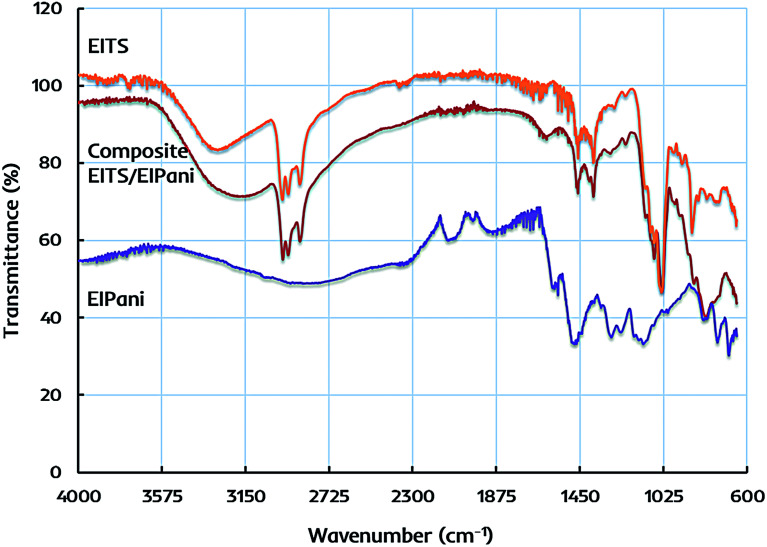
FTIR spectra of ethanol imprinted titania sol (EITS), ethanol imprinted polyaniline (EIPani), and dual-imprinted EITS–EIPani composite receptor layer.

The primary structural characteristics of receptor layers are studied by FTIR spectroscopy to identify the functional groups in EITS, EIPani, and the respective EITS–EIPani composite films. The spectra are recorded with Cary 630 diamond ATR (attenuated total reflection) accessory. Fig. 3 shows the representative FTIR spectra of these receptor materials. In case of EITS, a broad and intense band in 3550–3000 cm^−1^ region shows the presence of hydroxyl groups originating from the template, *i.e.* ethanol, hydrolyzed butyl, and terminal groups of titania sol. The transmittance peaks at 2960, 2931 and 2874 cm^−1^ indicate C–H stretching vibrations corresponding to CH_2_ and CH_3_ groups of ethanol and hydrolyzed butyl group of titanium butoxide. Furthermore, a strong peak near 1040 cm^−1^ shows C–O stretching vibrations. A band near 1460–1380 cm^−1^ relates to symmetric deformation of CH_3_ groups. Peaks in the range of 750–700 cm^−1^ indicate Ti–O–Ti stretching in titania that represents the formation of sol network.^47–49^

FTIR spectrum of EIPani exhibits two main peaks at 1560 cm^−1^ and 1485 cm^−1^, which represent the stretching vibration of C=C quinoid and C=C benzenoid rings of EIPani thus, indicating the formation of polyaniline. Moreover, peaks at 1290 cm^−1^ and 1126 cm^−1^ are due to C–N stretching of benzoid and quinoid forms.^50–52^ Finally, peaks in 860–680 cm^−1^ region indicate aromatic C–H bending vibrations. FTIR spectrum of EITS–EIPani composite contains the key structural features of both EITS and EIPani, as shown in Fig. 3. Additionally, it can be observed that 3550–3000 cm^−1^ region becomes more broad, intense with a peak shift to slightly lower value compared to EITS, which shows the hydrogen bonding interactions between N–H groups of polyaniline, and O–H groups of titania sol and the template. Since ethanol is used as the template for the synthesis of EITS and EIPani, it develops strong hydrogen bonding interactions with the matrix.^53^ Thus, the dual-imprinted composite receptor layer could retain high chemical and structural affinity toward ethanol in complex mixtures. Furthermore, based on non-covalent interactions, the developed imprinted sites in receptor layer could reversibly accommodate target analyte *i.e.* ethanol which would be beneficial for faster recovery.

### Surface characterization

**Fig. 4:**
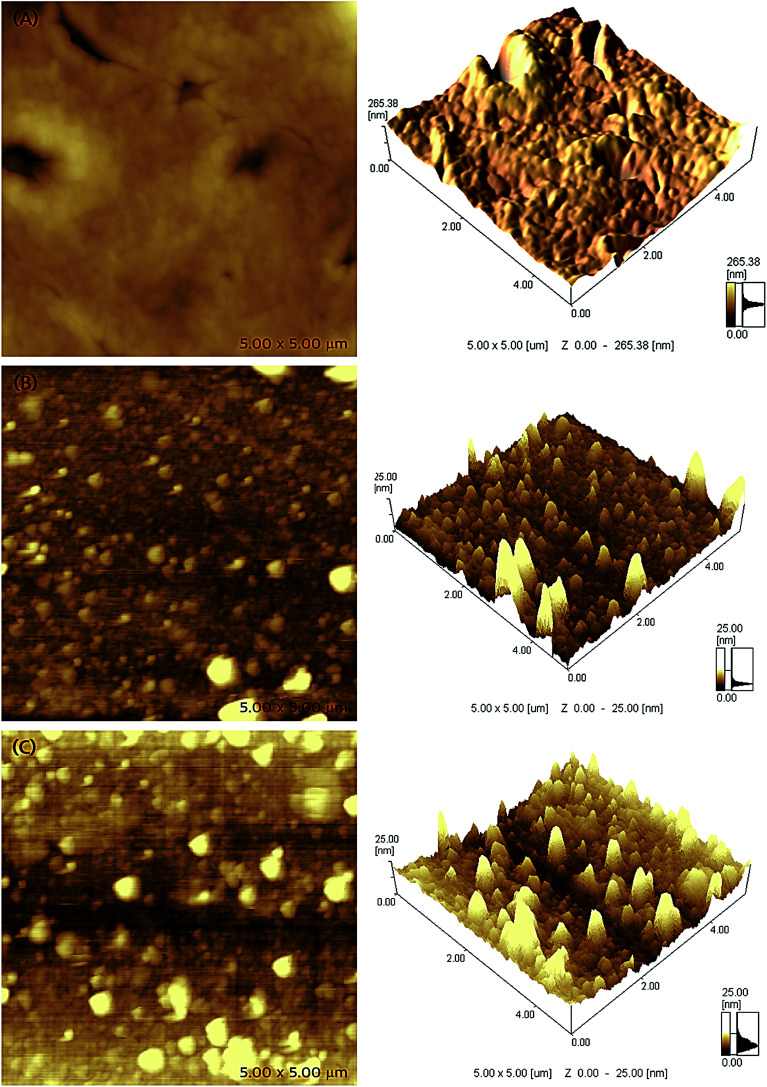
Two- and three-dimensional surface atomic force micrographs of (a) ethanol imprinted titania sol (EITS), (b) ethanol imprinted polyaniline (EIPani), and (c) dual-imprinted EITS–EIPani composite receptor layers spin coated on the surface of interdigital capacitor (IDC) transducers and vacuum dried at 25 °C.

The microstructure and surface morphology of different receptor layers are compared through AFM microscopic images, which are shown in Fig. 4. The two- and three-dimensional AFM micrographs of EITS film shown in Fig. 4a exhibit uniform glassy surface morphology with few defects or micro-cracks, which may be formed by the evaporation of solvent/template molecules. AFM images of EIPani films are shown in Fig. 4b, which indicate that polymer nanoparticles are evenly distributed in a thin film. The radius of EIPani nanoparticles lies in the range of 42–90 nm. Albeit a few agglomerates with a mean radius of 125 nm are found in these images, the average EIPani nanoparticles radius is approximately 62 nm.

The surface morphology of EITS–EIPani composite formed by mixing EIPani nanoparticles in EITS through ultrasound sonication, and spin coated on IDC transducers to form uniform dual-imprinted composite films is shown in Fig. 4c. Both two- and three-dimensional micrographs demonstrate homogeneous distribution of EIPani nanoparticles in EITS. It is obvious that EIPani particles are embedded in EITS with their radii in the range of 80–240 nm. The mean particle radius increases from 62 nm for pristine EIPani nanoparticles to 190 nm for EIPani particles embedded in EITS due to agglomeration in viscous EITS medium. However, the dispersion of EIPani nanoparticles in EITS matrix is consistently uniform. Furthermore, three-dimensional AFM images of pristine EIPani and EITS–EIPani composite films show comparable surface roughness.

### Metrological sensor characteristics

**Fig. 5:**
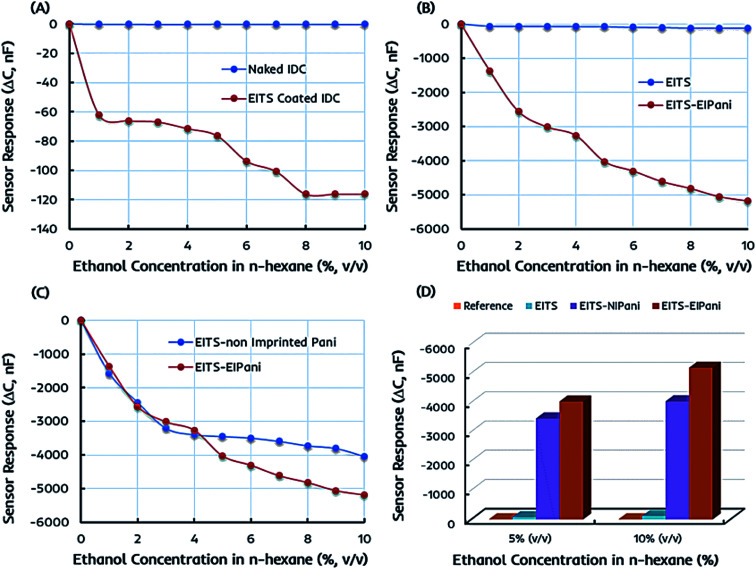
A comparison of the sensor responses of miniaturized interdigital capacitor (IDC) devices toward different concentrations of ethanol in *n*-hexane: (a) EITS coated IDC *vs.* the reference, *i.e.* naked IDC; (b) pristine EITS *vs.* dual-imprinted EITS–EIPani composite film; (c) single-imprinted EITS–NIPani *vs.* dual-imprinted EITS–EIPani composite film; and (d) the sensor responses of different receptors (EITS–EIPani, EITS–NIPani, EITS) and reference channel toward 5 and 10 vol% ethanol solutions.

The preliminary sensor measurements are performed in 1–10 vol% standard ethanol solutions formed by mixing anhydrous ethanol in *n*-hexane to determine the metrological sensor characteristics such as sensitivity of different receptors. The change in capacitance of the sensitive layer is monitored and plotted against different ethanol concentrations. Fig. 5a represents the shift in capacitance as sensor response of EITS-coated IDC device and the response of naked or uncoated IDC device as the reference. For EITS-coated IDC device, the capacitance value changes as a function of ethanol concentration, whereas there is no noticeable shift in the capacitance of naked IDC when exposed to same ethanol solutions. It is obvious that EITS film is capable of distinguishing ethanol molecules in *n*-hexane solution and absorbing them due to the presence of template analogous interaction sites generated by the imprinting procedure.

The capacitive sensor fundamentally translates the changes in capacitance of the active layer caused by the changes in its dielectric constant, for instance, because of the variations in the polarization properties of atoms and/or molecules within the active layer.^54,55^ This could be caused by external perturbations such as the molecular interactions between the polar organic compounds and the active layer. Vello *et al.*^56^ has already demonstrated that the capacitance of active layer changes with the polarity and/or dielectric properties of the analyte of interest. Thus, the sensitivity or sensor response of EITS–EIPani composite layer can be attributed to the interactions of ethanol (analyte) molecules with the composite layer. Ethanol is polar and has a higher dielectric constant compared to *n*-hexane and gasoline. Thus, ethanol–hexane mixtures and gasohol fuel blends have different dielectric properties compared to pure hexane and gasoline samples. Since, EITS–EIPani composite layer is permeable to ethanol and interactions with relatively polar ethanol molecules result in greater change in capacitance.

Ethanol itself is a relatively small molecule with low molecular weight therefore, we used ethanol as the solvent for synthesizing titania sol as it would lead to generate adapted interaction sites for ethanol recognition. A similar strategy has been used for developing sensor coatings where analyte to be measured was used as template and solvent at the same time to generate imprinting effects in polymer network.^53,57^ Furthermore, ethanol has a dielectric constant value of about 24.55 at 25 °C, which is much higher than that of *n*-hexane, *i.e.* 1.88 at 25 °C. Thus, the incorporation of ethanol in EITS receptor film results in decreased capacitance. This shift in capacitance is greater at higher concentration of ethanol in *n*-hexane. The naked IDC (*i.e.* reference) does not have any specific receptor layer coating; therefore, it does not show any significant sensor signal.

In the next phase, the sensor responses of dual-imprinted EITS–EIPani composite film are measured for different ethanol concentrations. The comparative capacitance shift of pristine EITS and dual-imprinted EITS–EIPani composite films against different ethanol concentrations is shown in Fig. 5b. EITS is the supporting matrix to immobilize EIPani nanoparticles and the resulting composite film is prepared with intent to improve device sensitivity and specificity. It is observed that sensor response has substantially improved by embedding EIPani nanoparticles in EITS matrix. Thus, it is obvious that the recognition properties of the receptor layer are further enhanced by the formation of EITS–EIPani composite. The dual-imprinted EITS–EIPani composite film exhibits higher sensitivity toward ethanol due to the collaborative imprinting effect of its constituents.

To further investigate the effect of imprinting, non-imprinted polyaniline (NIPani) nanoparticles are also embedded into EITS matrix to prepare single-imprinted EITS–NIPani composite film. Fig. 5c compares the sensor responses of single-imprinted EITS–NIPani and dual-imprinted EITS–EIPani composite films toward different ethanol solutions. Albeit, at lower concentrations of ethanol, *i.e.* 1–3 vol% ethanol in *n*-hexane, the sensor responses of both receptors are comparable. However, at higher concentrations of ethanol, a sizeable difference in the sensor response of two devices is recorded. The sensor response of single-imprinted EITS–NIPani composite film is saturated above 4 vol% ethanol in *n*-hexane, while dual-imprinted EITS–EIPani composite film demonstrates a gradual shift in capacitance at higher concentrations of ethanol. It also suggests that the imprinting of ethanol in EIPani nanoparticles greatly improves the sensitivity. Fig. 5d exhibits a summary of ethanol sensing measurements with different types of receptors. It is obvious that devices coated with dual-imprinted EITS–EIPani composite film exhibit the highest sensitivity toward ethanol in *n*-hexane mixtures.

**Fig. 6:**
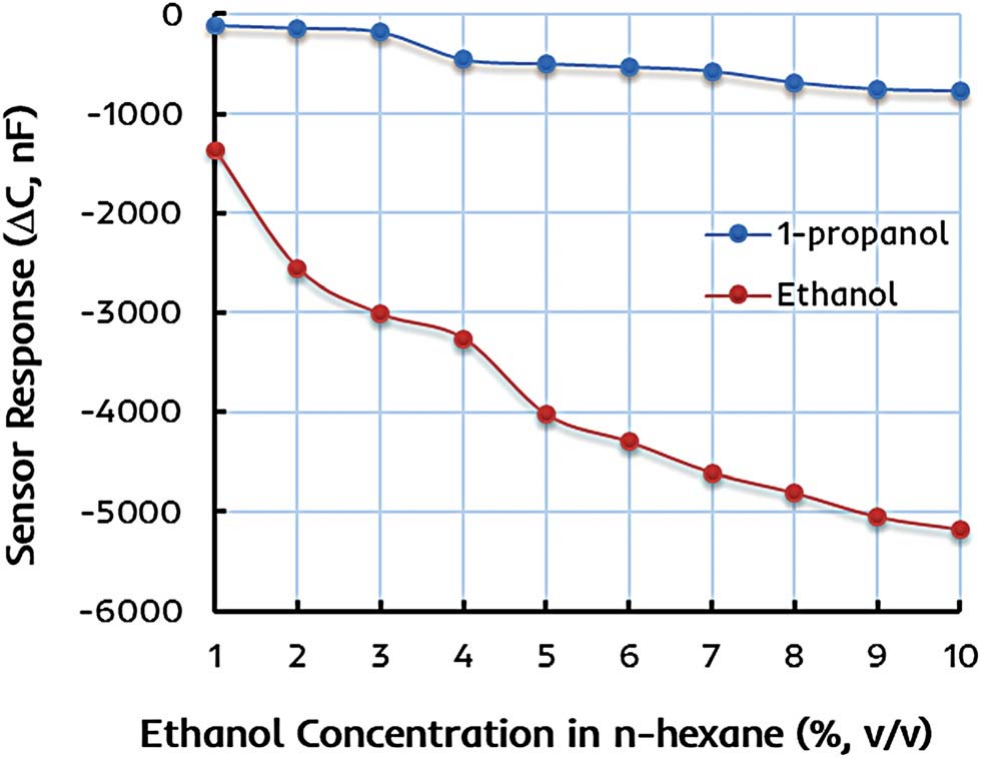
The sensor response of an interdigital capacitor (IDC) coated with dual-imprinted EITS–EIPani composite film toward different concentrations of ethanol and 1-propanol in *n*-hexane. The device exhibits strong liking and higher response for ethanol molecules due to the imprinting phenomenon.

The selectivity and specificity of the dual-imprinted composite film are determined by exposing it to the similar concentrations of 1-propanol in *n*-hexane solution. Fig. 6 shows the sensor response of EITS–EIPani composite film toward different concentrations of ethanol and 1-propanol in *n*-hexane. Understandably, the device coated with EITS–EIPani composite exhibit markedly high sensor response toward ethanol, while a small shift in capacitance is demonstrated at higher concentrations of 1-propanol. This is because dual-imprinted EITS–EIPani composite film contains highly adapted interaction sites for ethanol reincorporation thanks to the imprinting effect. Using ethanol as template and solvent for the synthesis of EITS and EIPani and its subsequent removal from the receptor layer leads to the formation of size, shape, and functionality specific cavities for ethanol recognition, and that is a suitable method for generating selective receptors.^57^ Furthermore, the selectivity of the composite layer toward ethanol in comparison with propanol is attributed to the inherent difference in polarity and dielectric properties of the two alcohols, *i.e.* ethanol is more polar and has a higher dielectric constant compared to 1-propanol, as well as to the imprinting effect, *i.e.* ethanol being the template molecule has a greater chance to interact with and diffuse into the active layer resulting in higher shift in capacitance.

### Sensor performance in gasohol fuel blends

**Fig. 7:**
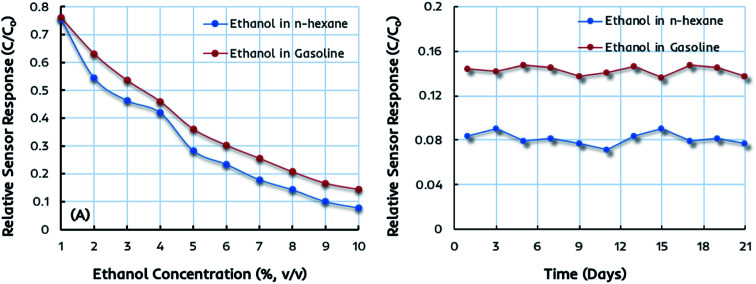
(A) A comparison of the relative sensor response of an interdigital capacitor (IDC) coated with dual-imprinted EITS–EIPani composite receptor toward different concentrations of ethanol in *n*-hexane and real-time gasohol fuel blends corresponding to 1–10 vol% ethanol (E1–E10). The comparable sensor performance displays the practical applications of this device in complex mixtures and gasohol fuel blends. (B) The sensor response of EITS–EIPani composite sensor toward 10 vol% ethanol in *n*-hexane and real-time gasohol fuel blend showing stability of the device during three weeks of testing. The sensor exhibits about 95% response retention after three weeks.

The miniaturized IDC devices coated with dual-imprinted EITS–EIPani composite as the receptor are finally tested in complex mixtures, *i.e.* the gasoline–ethanol (gasohol) fuel blends with 1–10 vol% ethanol. These gasohol blends, labeled as E1–E10, are prepared according to the ASTM standards for laboratory analysis and stored in airtight containers before sensor measurements. Fig. 7a shows the relative sensor response of an IDC device coated with dual-imprinted EITS–EIPani composite receptor toward different gasohol blends (E1–E10), and the responses are compared with standard ethanol solutions in *n*-hexane. Clearly, both sensor measurements are closely related to each other showing an increase in sensor response as a function of ethanol concentration. It shows that these miniaturized electronic devices combined with a suitable and specific receptor layer offer excellent sensitivity toward ethanol not only in pure *n*-hexane solutions but in complex mixtures containing gasoline. Fig. 7b shows the sensor response stability after three weeks of testing the device in 10% (v/v) ethanol in *n*-hexane and gasohol fuel blends. The EITS–EIPani composite sensor shows excellent stability with 95–96% retention of the sensitivity after three weeks of testing. Furthermore, the response does not fluctuate greatly during the testing period. These measurements also demonstrate the potential and capability of these devices in monitoring variable ethanol concentrations in blended fuels. Indeed, there is a great possibility of using these electronic sensors as onboard fuel quality control platforms due to their small size, fast response, ease of fabrication, cost-effectiveness, and practical performance in terms of instantaneous detection of ethanol in gasohol blends and other biofuels.

### Comparison of sensor performance

The performance of developed sensor for ethanol monitoring in gasohol fuel blends is compared with already reported electronic sensor devices as shown in Table 2. The comparison is made in terms of transducer design including device fabrication geometry and details, chemical sensor coatings, ethanol detection in liquid or gas phase, lowest tested concentration of ethanol in fuel, selectivity and applicability to real time samples.

**Table 2:** Comparison of developed sensor setup with already reported detection strategies for estimation of ethanol in gasohol fuel blends

Sensor details	Rocha *et al.*^58^	Benvenho *et al.*^59^	Present work
Transduction principle	Electronic sensor	Electronic sensor	Electronic sensor
Nature of electrodes	Coaxial stainless steel electrodes	Tin-coated copper interdigitated electrodes	Gold-coated copper interdigitated electrodes
Geometry of transducer	32.4 × 6.0 mm^2^	23 × 9 mm^2^	10 × 9 mm^2^
Gap between electrodes	—	0.20 mm	0.13 mm
Chemical sensor coating	—	Poly[(2-bromo-5-hexyloxy-1,4-phenylenevinylene)-*co*-(1,4-phenylenevinylene)] doped with dodecylbenzenesulfonic acid	Ethanol-imprinted poly-aniline dispersed in ethanol-imprinted titania sol
Ethanol detection phase	Liquid phase	Vapour phase	Liquid phase
Operating temperature	−10 to 40 °C	25 °C	25 °C
Lowest tested concentration	10%	5%	1%
Selectivity against 1-propanol	Not tested	Not tested	Good
Applicability to real samples	Good	Good	Good

From this comparison, it can be seen that our developed electronic sensor is capable of determining ethanol in gasohol samples as low as 1% and furthermore, it offered high selectivity when tested against 1-propanol. In terms of transducer design, the gold coated electrodes of the developed sensor is suitable in designing rugged devices having good stability in corrosive medium *i.e.* gasoline. Furthermore, it is already studied that titania based sensor coatings offer adequate chemical and thermal stability in corrosive medium.^60^ Comparing to other sensor devices, the miniaturized design of transducer is suitable for developing onboard fuel quality monitoring system.

## Conclusion

In this study, a miniaturized electronic device with an active surface area of 0.9 cm^2^ is developed by screen printing interdigital transducer electrodes and coating them with a selective receptor layer to instantly detect ethanol in *n*-hexane and gasohol fuel blends. The receptor is composed of a dual-imprinted EITS–EIPani composite film fabricated on the interdigital capacitor (IDC) by spin coating. These devices are inexpensive, small, easy to fabricate, and do not require extensive laboratory procedures. An IDC works as an efficient transducer in combination with the receptor, thus translating slight shift in capacitance into an electrical signal when the receptor layer interacts with ethanol molecules. The devices exhibit instant signals upon exposure to ethanol solutions and gasohol E1–E10 fuel blends, and the sensor responses increase as a function of ethanol concentration. The imprinting approach induces higher sensitivity and greater specificity toward ethanol in dual-imprinted composite films due to size, shape, and functionality specific recognition characteristics. The relative sensor response of these devices toward gasohol fuel blends is promising and demonstrates their practical applications in monitoring real-time ethanol concentration in fuel. Furthermore, the smaller size makes IDCs suitable for developing miniaturized onboard sensing platforms. In future, the optimization of receptor material composition and device design may lead to the development of reliable electronic sensor chips for precise and accurate onboard analysis of gasohol fuel blends and other biofuels.

## Conflicts of interest

The authors declare no competing financial interest.

## Supplementary Material
